# Supplementary material for: Phytochemical Cue for the Fitness Costs of Herbicide-Resistant Weeds
Source: Plants (Basel). 2023 Sep 2;12(17):3158. doi: 10.3390/plants12173158 (PMC10490342; doi:10.3390/plants12173158)
Supplement: Supplementary file 1 [file plants-12-03158-s001.zip › plants-2546767-supplementary.pdf]

plants

Supplementary materials

Phytochemical cue for the fitness costs of herbicide-resistant weeds

Hong-Yu Li[1], Yan Guo [1], Bo-Yan Jin [1], Xue-Fang Yang[2,*] and Chui-Hua Kong [1,*]

This supplementary material was prepared to add the readers more details, for which there was not enough space in the main manuscript, about:

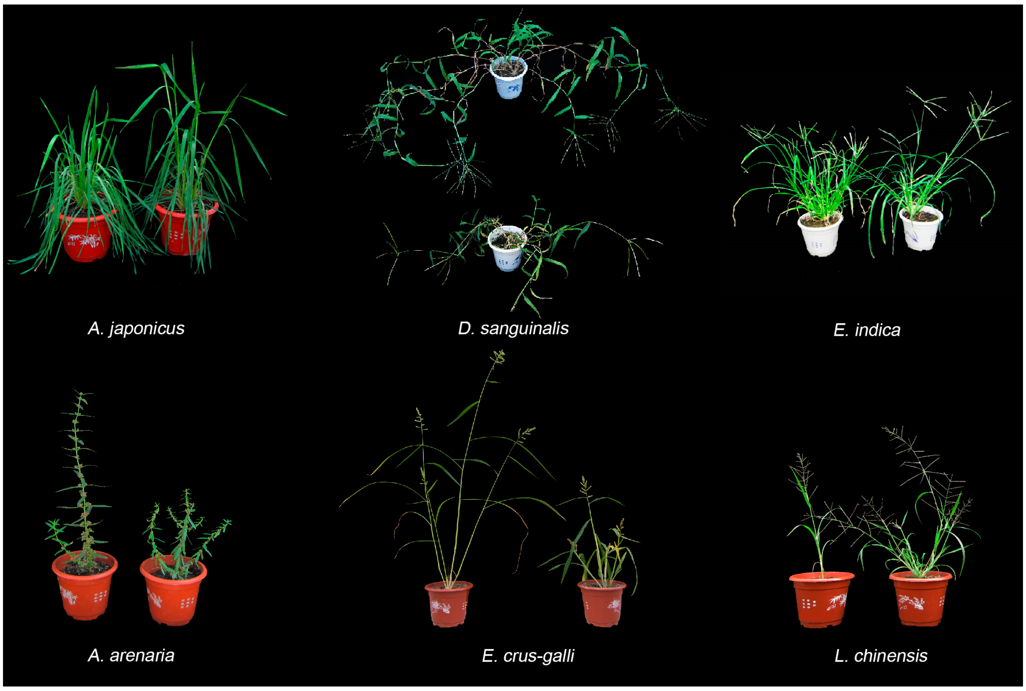

Figure S1. Aboveground morphology of herbicide-resistant and -susceptible weeds at the flowering stage. The left or above are herbicide-resistant biotypes and the right or below are the susceptible counterparts within a weed species.

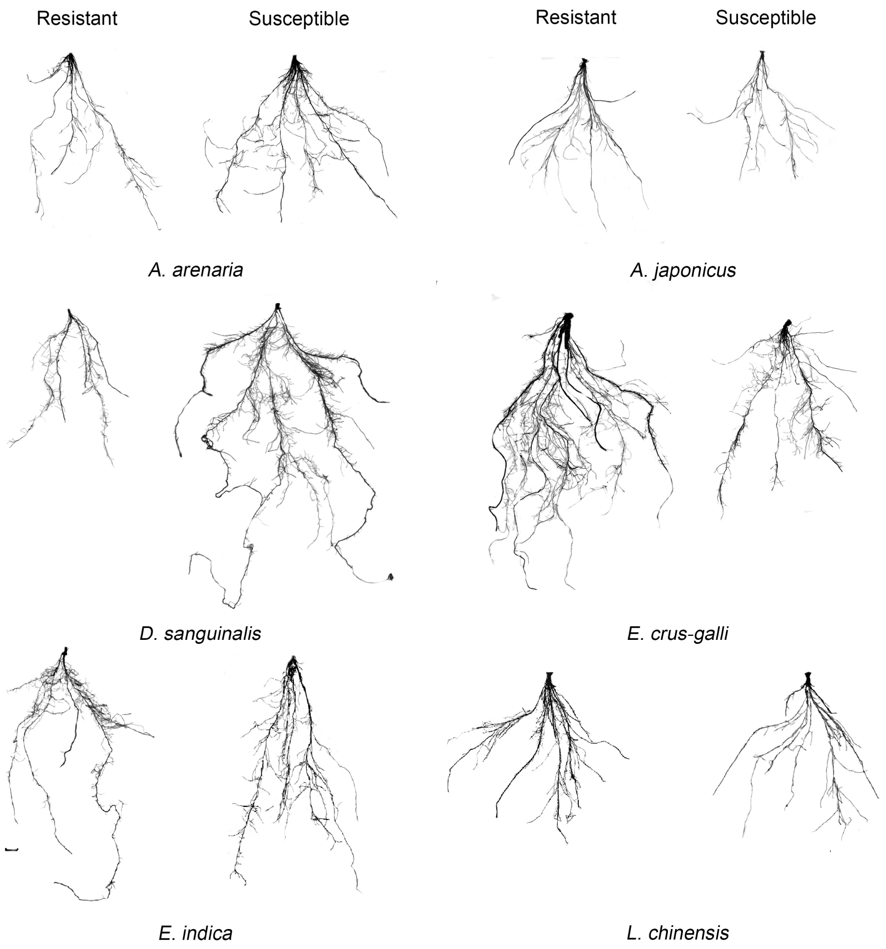

Figure S2. Root morphology of herbicide-resistant and -susceptible weeds. The left is herbicide-resistant biotypes and the right is the susceptible counterparts within a weed species.

Table S1. Plant height and biomass of herbicide-resistant and -susceptible weeds at the seedling and tillering stages

Stage	Weeds	Herbicide	Biotypes	Height (cm)	Shoot biomass (mg)	Root biomass (mg)	Root/Shoot (biomass)
Seedling	*Alopecurus japonicus*	fenoxaprop-p-ethyl	resistant	11.300±0.100b	6.100±0.802a	4.933±0.669a	0.808±0.016a
			susceptible	14.867±0.067a	6.233±0.348a	3.367±0.260a	0.540±0.032b
	Digitaria sanguinalis	glyphosate	resistant	18.933±2.331a	249.000±50.090b	33.333±7.965b	0.132±0.005a
			susceptible	27.433±1.071a	1136.667±166.859a	84.000±14.731a	0.078±0.022b
	Eleusine indica	glyphosate	resistant	3.567±0.167a	37.433±2.501a	28.833±1.695a	0.772±0.019a
			susceptible	2.733±0.260b	23.067±2.21b	18.367±4.108b	0.776±0.109a
	Ammannia arenaria	bensulfuron-methyl	resistant	8.767±0.869a	39.467±2.919b	3.300±0.404b	0.084±0.012a
			susceptible	10.233±0.353a	66.867±8.650a	7.667±1.132a	0.115±0.011a
	Echinochloa crus-galli	metamifop	resistant	37.200±0.379a	91.667±3.671a	24.467±1.601a	0.267±0.013a
			susceptible	29.733±1.235b	55.600±6.295b	16.100±1.779b	0.295±0.036a
	Leptochloa chinensis	cyhalofop-butyl	resistant	7.067±0.233a	6.767±0.639a	2.100±0.265a	0.309±0.013a
			susceptible	6.533±0.406a	8.900±0.651a	2.400±0.513a	0.267±0.048b
Tillering	*Alopecurus japonicus*	fenoxaprop-p-ethyl	resistant	20.567±0.617a	82.4±15.435b	17.6±3.361b	0.214±0.009a
			susceptible	22.667±0.333a	178.9±28.016b	40.9±7.845a	0.226±0.007a
	Digitaria sanguinalis	glyphosate	resistant	56.633±2.17a	826.6±48.579b	157.733±7.792b	0.191±0.006a
			susceptible	66.933±3.325a	2646.233±117.986a	351.4±39.676a	0.134±0.019b
	Eleusine indica	glyphosate	resistant	18±1.756a	280.633±29.74a	58.233±6.629b	0.216±0.04b
			susceptible	18.333±1.424a	277.333±23.398a	90.633±7.835a	0.332±0.045a
	Ammannia arenaria	bensulfuron-methyl	resistant	14.57±0.591b	274.333±10.145b	28.833±2.154b	0.106±0.012a
			susceptible	16.067±0.437a	420.3±11.778a	50.233±2.82a	0.12±0.007a
	Echinochloa crus-galli	metamifop	resistant	88.967±0.939a	5356.667±886.648a	613.333±60.645a	0.118±0.011b
			susceptible	61.5±2.066b	696.667±107.134b	183.333±17.638b	0.274±0.046a
	Leptochloa chinensis	cyhalofop-butyl	resistant	21.567±1.027a	79.633±3.656a	17.867±0.338a	0.225±0.007a
			susceptible	20.267±0.717a	69.067±4.119b	15.833±0.857a	0.232±0.025b

Values are means ± SE. Data with different letters indicate a significant different at $P < 0.05$, according to one-way ANOVA, followed by Tukey HSD test

Table S2. Eigenvalues and variance contributions of different resistant and sensitive biotypes of weed factors

Component	Initial Eigenvalue			Extract the sum of squared loads		
	Total	Percent Variance	Accumulate%	Total	Percent Variance	Accumulate%
1	6.427	29.213	29.213	6.427	29.213	29.213
2	5.274	23.972	53.184	5.274	23.972	53.184
3	3.919	17.813	70.997	3.919	17.813	70.997
4	2.069	9.407	80.404	2.069	9.407	80.404

Table S3. Component load matrix (PC1-4) and principal component coefficient (Y1-4) of each characteristic index of weeds with different resistant and susceptible biotypes

Index	PC1	F1	PC2	F2	PC3	F3	PC4	F4
Height	0.438	0.173	0.681	0.297	-0.262	-0.133	0.336	0.233
AG biomass	0.679	0.268	0.413	0.180	-0.265	-0.134	0.454	0.315
BG biomass	0.602	0.238	0.086	0.037	0.171	0.086	0.698	0.485
Root length	0.695	0.274	0.590	0.257	-0.195	-0.098	-0.100	-0.069
Root surface area	0.731	0.288	0.372	0.162	-0.265	-0.134	0.018	0.012
Root volume	0.802	0.316	0.486	0.212	-0.038	-0.019	0.034	0.024
Photosynthetic rate	0.497	0.196	-0.704	-0.306	0.271	0.137	0.389	0.270
Stomatal conductance	0.658	0.260	-0.597	-0.260	0.301	0.152	0.057	0.040
Transpiration rate	0.653	0.258	-0.613	-0.267	0.335	0.169	0.011	0.008
Chlorophyll	-0.241	-0.095	0.573	0.250	-0.417	-0.211	-0.369	-0.257
Shoot CAT	-0.562	-0.222	0.297	0.129	0.615	0.311	0.279	0.194
Root CAT	0.496	0.196	-0.364	-0.159	0.699	0.353	-0.268	-0.186
Shoot SOD	-0.064	-0.025	0.443	0.193	0.714	0.361	-0.029	-0.020
Root SOD	0.245	0.097	0.505	0.220	0.480	0.242	-0.564	-0.392
Shoot MDA	-0.384	-0.152	0.501	0.218	0.604	0.305	0.328	0.228
Root MDA	-0.317	-0.125	0.468	0.204	0.685	0.346	0.344	0.239
Shoot phenols	-0.332	-0.131	0.599	0.261	-0.296	-0.150	0.274	0.190
Root phenols	-0.345	-0.136	0.379	0.165	0.090	0.045	-0.003	-0.002
Shoot (–)-loliolide	-0.609	-0.240	-0.306	-0.133	-0.410	-0.207	0.199	0.138
Root (–)-loliolide	-0.841	-0.332	0.163	0.071	0.057	0.029	0.171	0.119
Days to flowering	-0.158	-0.062	-0.468	-0.204	-0.677	-0.342	0.305	0.212
Seed production	0.620	0.244	0.610	0.266	-0.160	-0.081	-0.177	-0.123

Table S4. Principal component scores of herbicide-resistant and -susceptible weeds

Weeds	Biotypes	Y1	Y2	Y3	Y4	Y
	Resistant	-0.878	-1.495	-0.421	-1.837	-0.863
Ammannia arenaria	Susceptible	-1.817	-0.602	-1.149	-2.203	-1.087
	Resistant	1.462	-2.623	-0.358	-0.475	-0.310
Alopecurus japonicus	Susceptible	-0.910	-2.899	-2.820	0.704	-1.397
	Resistant	-1.643	-0.383	-1.650	0.988	-0.773
Digitaria sanguinalis	Susceptible	1.992	1.745	-2.858	2.544	0.730
	Resistant	4.006	4.532	-0.552	-0.944	2.070
Echinochloa crus-galli	Susceptible	0.295	2.344	0.615	-1.664	0.601
	Resistant	2.904	-1.955	3.452	1.418	1.128
Eleusine indica	Susceptible	2.238	-2.036	2.304	-0.161	0.561
	Resistant	-4.867	2.155	2.417	1.290	-0.353
Leptochloa chinensis	Susceptible	-2.783	1.217	1.021	0.341	-0.307

Table S5. Herbicide-resistant weeds and their resistance mechanisms

	Herbicide-resistant weeds	Resistance type	Mutation sites	Location of origin	Source
**Wheat fields**	Fenoxaprop-p-ethyl-resistant *Alopecurus japonicus*	Non-target resistance	None	Chuzhou, Anhui Province, China (32°83′80.48″N, 119°4′74.44″E)	Bi et al., (2016)
	Glyphosate-resistant *Digitaria sanguinalis*	Non-target resistance	None	Changde, Hunan Province, China (29°24′41.11″ N, 112°10′15.46″E)	Li et al., (2016)
	Glyphosate-resistant *Eleusine indica*	Target resistance	Ser102-Ile	Chengdu, Sichuan Province, China (30°32′48.7′′N,103°54′53.2′É)	Chen et al., (2017)
**Paddies**	Bensulfuron-resistant *Ammannia arenaria*	Non-target resistance	None	Suzhou, Jiangsu Province, China (31°18′8.17″ N, 120°37′52.75″E)	Zhang et al., (2020)
	Metamifop -resistant *Echinochloa crus-galli*	Non-target resistance	None	Huai'an, Jiangsu Province, China (33°35′51.03″N, 119°01′16.55″E)	Li et al., (2015）
	Cyhalofop-resistant *Leptochloa chinensis*	Target resistance	Trp1999-Ser	Nanjing, Jiangsu Province, China (31°59'08"N, 118°25'55"E)	Peng et al., (2020)

Bi, Y.L.; Liu, W.T.; Guo, W.L.; Li, L.X.; Yuan, G.H.; Du, L.; Wang, J.X. Molecular basis of multiple resistance to Accase- and ALS-inhibiting herbicides *Alopecurus japonicas* from China. *Pestic. Biochem. Physil*. **2016**, *126*, 22-27.

Chen, J.C.; Huang, H.J.; Wei, S.H.; Huang, Z.F.; Wang, X.; Zhang, C.X. Investigating the mechanisms of glyphosate resistance in goosegrass (*Eleusine indica* (L.) Gaertn.) by RNA sequencing technology. *Plant J*. **2017**, *89*, 407-415.

Li, Y.; Zong, T.; Yang, H.N.; Bai, L.Y. Preliminary study on the resistance of *Digitaria sanguinalis* to glyphosate in cotton fields in the middle and lower reaches of the Yangtze river. *Cotton Sci*. **2016**, *28*, 300-306.

Li, Y.F.; Zhang, Z.C.; Yang, X.; Dong, M.C.; Zhang, B.; Han, J.Y. Susceptibility of weeds in *Echinochloa* to aryloxyphenoxypropionate herbicides and the mechanism. *Jiangsu J. Agric. Sci*. **2015**, *31*, 543-551.

Peng, Y.J.; Pan, L.; Liu, D.C.; Cheng, X.M.; Ma, G.L.; Li, S.F.; Liu, X.Y.; Wang, L.F.; Bai, L.Y. Confirmation and characterization of cyhalofop-butyl-resistant Chinese sprangletop (*Leptochloa chinensis*) populations from China. *Weed Sci*. **2020**, *68*, 253-259.

Zhang, J.S.; Liu, B.; Cai, X.Z.; Zhou, W.J.; Wang, H.F.; Lu, Q.; Zhou, G.J.; Liu, Y.G.; Liang, W.; Wang, S.; Zhu, J.W. Resistance and its resistant molecular mechanism of *Ammannia arenaria* to ALS inhibiting herbicides. *Chin. J. Pestic. Sci*. **2020**, *22*, 60.
